# Nanofluid Types, Their Synthesis, Properties and Incorporation in Direct Solar Thermal Collectors: A Review

**DOI:** 10.3390/nano7060131

**Published:** 2017-05-31

**Authors:** Wisut Chamsa-ard, Sridevi Brundavanam, Chun Che Fung, Derek Fawcett, Gerrard Poinern

**Affiliations:** 1 Murdoch Applied Nanotechnology Research Group, Department of Physics, Energy Studies and Nanotechnology, Murdoch University, Murdoch, WA 6150, Australia; wisut_sert@hotmail.com (W.C.-a.); sridevi.brundavanam@gmail.com (S.B.); fawcett@southwest.com.au (D.F.); 2 School of Engineering and Information Technology, Murdoch University, Murdoch, WA 6150, Australia; L.Fung@murdoch.edu.au

**Keywords:** nanofluids, solar thermal, energy conversion, thermal conductivity

## Abstract

The global demand for energy is increasing and the detrimental consequences of rising greenhouse gas emissions, global warming and environmental degradation present major challenges. Solar energy offers a clean and viable renewable energy source with the potential to alleviate the detrimental consequences normally associated with fossil fuel-based energy generation. However, there are two inherent problems associated with conventional solar thermal energy conversion systems. The first involves low thermal conductivity values of heat transfer fluids, and the second involves the poor optical properties of many absorbers and their coating. Hence, there is an imperative need to improve both thermal and optical properties of current solar conversion systems. Direct solar thermal absorption collectors incorporating a nanofluid offers the opportunity to achieve significant improvements in both optical and thermal performance. Since nanofluids offer much greater heat absorbing and heat transfer properties compared to traditional working fluids. The review summarizes current research in this innovative field. It discusses direct solar absorber collectors and methods for improving their performance. This is followed by a discussion of the various types of nanofluids available and the synthesis techniques used to manufacture them. In closing, a brief discussion of nanofluid property modelling is also presented.

## 1. Introduction

Global challenges facing the world today include increasing energy demands, greenhouse gas emissions, global warming and environmental degradation. Humanities insatiable demand for energy has never been satisfied and continues to escalate. Energy is an important thermodynamic quantity that defines the ability of a physical system to accomplish work. The efficient conversion of energy to perform useful work is a factor that contributes to the financial development and economic sustainability of a country. At present, global consumption (~86%) relies heavily on finite fossil fuel resources. Globally, fossil fuel consumption consists of petroleum products used in the transport sector (~32.6%), natural gas for heating (~23.7%) and coal (~30.0%) being used to generate electricity [1]. In spite of being a limited resource, the consumption of fossil fuels continues unabated and consequences such as global warming and environmental degradation are becoming more serious. Data forecasting the next 20 years has revealed the world population will increase by 1.5 billion, equating to a global population of around 8.8 billion people. During this period, annual global energy demand is expected to increase by 1.4%, with more than half of this demand resulting from increased electrical power generation [1]. The detrimental impact of spent fossil fuels on the environment is also expected to increase. Because of the hazardous impact, current research efforts are focused on developing clean, viable and renewable energy sources. Current estimates indicate renewable energy sources are expected to grow at a rate of 6.6% annually for the next 20 years. Resulting in renewable energy sources contributing to around 9% of the total global energy supply [1]. Therefore, considering the long-term global demand for energy and the imperative need to reduce the impact of global warming and environmental degradation, developing new and improving the efficiency of currently available renewable energy sources has become a major global challenge [2,3]. Bearing in mind the minor contribution made by renewable energy sources to the current global energy demand, there is significant opportunity to increase its contribution. Especially in the case of solar energy, which only accounts for around 0.33% of the current total energy mix [1]. Thus, increasing the renewable energy contribution is importance, since only renewable energy sources have the potential to alleviate global warming, decrease greenhouse gas emissions and reduce environmental degradation [4]. 

The Sun’s formation is estimated to have taken place around 4.6 billion years ago, and since then has driven climatic conditions and supported life on Earth. The Sun releases enormous amounts of energy (~63 MW/m^2^) that is radiated into space. However, because of Sun-Earth geometry and the distance between, the energy flux is dramatically reduced to around 1350 W/m^2^ (the so-called “Solar Constant”) before reaching the Earth’s atmosphere [5]. Around 30% of all sunlight showering the Earth’s upper atmosphere is reflected back into space. The remaining 70% passes through the atmosphere before being absorbed by land masses and oceans. Passage through the atmosphere filters out much of the harmful ultraviolet radiation, leaving only a small component in the near-ultraviolet. Importantly, atmospheric gases such as ozone (O_3_), carbon dioxide (CO_2_), and water vapor (H_2_O) are instrumental in attenuating the incoming solar irradiation. Attenuation is due to the bond energies of these gases being close to the incoming photon energy level. Most of the light reaching the Earth’s surface is distributed across the visible and near-infrared regions of the spectrum (0.25 < λ < 2.5 µm) [6]. Overall the estimated amount of solar energy absorbed by the atmosphere, land masses and oceans is around 3.85 million exajoules (EJ) per year. Interestingly, estimates indicate the hourly solar energy levels absorbed by the Earth are greater than humanities current annual global energy demand [6,7,8,9]. Daily around 1 kW/m^2^ of solar energy reaches Earth’s surface. Intriguingly, most forms of renewable energy, other than geo-thermal and tidal power, are dependent on energy from the sun. Accordingly, solar energy has become one of the most heavily investigated and exploited sources of renewable energy. Furthermore, solar energy is not only eco-friendly, but it can assist in reducing greenhouse gas emissions and mediate the effects of global warming. However, because of its relatively low energy density at ground level and its intermittent nature due to the Earth’s rotation, it must be collected and stored efficiently. 

The need to collect and store solar energy has broadly resulted in two solar energy conversion methods being developed. The first is photovoltaic and involves converting solar energy directly into electrical energy. The second is solar thermal and involves converting solar energy directly into thermal energy. In photovoltaic (PV) systems, incoming sunlight strikes a semiconductor material (crystalline silicon) and dislodges electrons in the material to cause the movement of electrons. The resulting electron flow generates the PV cells electrical output. However, only a small portion of incident solar energy (typically less than 20%) is transformed into electrical energy. The remaining incident energy increases the temperature of the PV cell and reduces its operational performance [9,10]. The favorable photovoltaic properties of crystalline silicon have resulted in the material being used in a wide variety of PV solar cell configurations for many years. However, the high cost and low availability of high-quality solar-grade crystalline silicon makes PV solar cells expensive. Less expensive amorphous silicon-based PV cells are currently used, but they have lower efficiencies and are prone to photo-degradation over time [10,11]. Alternatively, solar thermal systems are used in many industrial, commercial and domestic applications to supply energy for heating water, space heating and cooling, and at higher temperatures to drive power generating turbines [12]. Solar thermal collectors are designed to absorb and convert solar irradiation into thermal energy. The energy is then transferred to a working fluid (typically air, water or oil) contained within the collector’s structure. The circulating working fluid carries the energy away from the collector in the form of heat to be directly used or used to charge up a thermal storage tank [13]. There are two methods for collecting solar thermal energy. The first uses non-concentrating collector configurations, where the absorbing panel is the same surface area as the collector. The second method uses several reflecting surfaces to gather, concentrate and focus the solar irradiation onto a collector. In this configuration, the surface area of the collector is much smaller than the surface area of the reflectors. Concentrated solar thermal (CST) collectors are capable of achieving higher temperatures than non-concentrating configurations. The higher temperatures can induce phase changes in the working fluid (water to steam) that drive turbines to generate electricity [14,15]. In these applications, improving collector performance and conversion efficiencies has attracted considerable interest from solar energy researchers in recent years. Currently, many industrial processes and commercial operations require heat between 80 and 250 °C. Thus, making solar thermal absorption collectors operating in this temperature range ideal for these applications. However, these collectors have shortcomings such as low solar capture, poor heat transfer characteristics and collector heat losses [16]. Therefore, to overcome these shortcomings direct absorption solar collectors were developed. The advantage of using direct absorption solar collectors comes from incident solar irradiation being directly transferred into the working fluid. The working fluid then flows in either an open-loop configuration for low energy applications or in a closed-loop arrangement for high energy applications. In a closed-loop configuration, the working fluid flows through a heat exchanger circuit that physically separates the working fluid from a secondary fluid (i.e., potable water) circuit. Working fluids used in heat exchanger circuits have included water, ethylene glycol, water/ethylene glycol mixtures and a wide range of oils. Unfortunately, these fluids display low adsorptive properties over the solar spectrum range (0.25 < λ < 2.5 µm) [17,18]. Furthermore, the thermal properties of these working fluids are also poor when compared to metals like copper (Cu) and iron (Fe), which are used in the construction of collectors. For example, the thermal conductivity of Cu (398 Wm^−1^K^−1^) is roughly three orders of magnitude greater than water (0.608 Wm^−1^K^−1^). The large difference in thermal conductivity also occurs with other commercially available working fluids such as ethylene glycol, water/ethylene glycol mixtures and oils. Thus, recent research has focused on improving the thermal properties of working fluids. For example, the inclusion of small concentrations of suspended nanometer scale particles in conventional working fluids can enhance their thermal properties. Accordingly, the present review surveys the various types of solar collectors in current use including both low-temperature systems (non-concentrating collectors) and high-temperature systems (concentrating collector). Then investigates recent developments in formulating a new class of engineering fluids containing small concentrations of suspended nanometer-scale particles. The presence of dispersed particles (metals, metal oxides and carbons) in working fluids can significantly improve their heat transfer characteristics. The preparation and stabilization of several nanofluid formulations, including carbon-based materials, are discussed at length. This is followed by a discussion of mathematical modelling used to predict thermal conductivity enhancements reported in the literature. The review concludes by discussing the performance of several nanofluid formulations in direct absorption solar collectors. 

## 2. Solar Thermal Collector Types

### 2.1. Solar Collectors

There are several types of solar thermal collectors currently available in the market place. The collectors are classified by their solar concentration ratio and tracking motion [13,19]. Non-concentrating collectors have a concentration ratio of around one and operate at temperatures ranging from 30 to 240 °C. Typically, non-concentrating collectors are permanently fixed in position and do not track the sun, but instead, rely on their initial orientation. Thus, in the northern hemisphere they face south and in the southern hemisphere face north. Concentrating solar collectors are designed to track the Sun’s position during the day and can use either single-axis or two-axis tracking configurations. Concentration ratios can range from 10 to 1500, have high radiation fluxes and can achieve temperatures as high as 2000 °C as seen in Table 1 below. The following sections discuss the various types of collectors currently in use around the world. 

### 2.2. Non-Concentrating Solar Collectors

The most common non-concentrating solar thermal energy converter is the flat-plate collector (FPC). This type of collector is extensively used for water-heating and space-heating applications in homes [28]. FPCs are specifically designed to operate in favorable climates that are warm and sunny. They are roof mounted, orientated and fixed in position for optimal solar exposure. Typically, a FPC consists of an insulated box fitted with a glass window or similar material with high transmission properties [29]. Within the box is an absorber, which consists of plates and tubes treated with a dark-colored solar selective coating to promote solar absorption [30]. Most of the solar energy entering the FPC assembly is absorbed by the plate-tube assembly. The resulting thermal energy is transferred to a circulating working fluid contained within the tubes. The temperature of the working fluid (usually water), rarely exceeds 80 °C in these collectors. Thus, making them ideal for supplying domestic hot water [20]. However, the operational performance of FPCs is significantly reduced during adverse weather events. For example, long periods of cold, cloudy and windy days can greatly reduce collector performance and during these periods additional electrical or gas heating is needed. An alternative type of non-concentrating collector currently available is the evacuated tube collector (ETC). An ETC consists of a central fluid channel contained within a vacuum-sealed tube assembly. The tube assembly consists of two concentric glass tubes, with the space between the two tubes being evacuated to reduce convection and conduction losses from the inner tube that contains the working fluid [31]. The effective suppression of convection by the vacuum envelope means the ETC can operate at much higher temperatures than conventional FPCs. The central heating tubes outer surface is also treated with optically desirable coatings designed to absorb as much energy as possible, which is then transferred to the working fluid [21]. The heating tubes are connected to a manifold system that permits the circulation of the working fluid in a similar manner to FPCs. Another permanently fixed solar collector is the compound parabolic collector (CPC). This type of collector consists of multiple internal reflecting parabolic surfaces that direct solar energy to an absorber tube located at the bottom of the collector assembly [22]. Thus, CPCs are capable of receiving large amounts of diffuse radiation without the need for sun-tracking. A further type of solar thermal absorber is the cylindrical tube collector. This collector consists of a cylindrical glass tube that forms the solar energy receiver and a central absorbing tube that carries the working fluid. The function of the outer glass cylinder is the same as the transparent cover found in many conventional solar panels. That is, reduce reflectivity and promote transmission of solar energy to the central tube. The cylindrical glass assembly is evacuated to avoid vapor build up on internal surfaces that would otherwise reduce energy transfer. The surface of the central collector tube is coated with a dark-colored solar selective material that promotes maximum solar absorption, which in turn promotes higher temperatures in the working fluid. The working fluid flow rate is regulated by valves to optimize maximum energy transfer [32].

### 2.3. Concentrating Solar Collectors 

**Figure 1:**
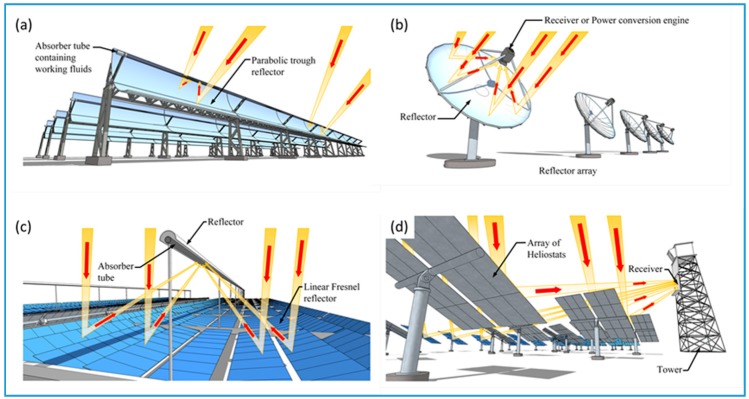
A selection of concentrating solar collectors styles adapted from the available literature: (**a**) parabolic trough collector; (**b**) parabolic dish collector; (**c**) linear Fresnel collector, and (**d**) heliostat field collector [14,23,26,33].

Concentrating solar collectors incorporate sun tracking technology to maintain the delivery of concentrated and focused solar radiation to the collector. The four main types of concentrating collectors are: (1) linear Fresnel reflectors; (2) parabolic troughs; (3) parabolic dishes, and (4) heliostats collectors. Linear Fresnel collectors (FLC) consist of either long slightly curved mirrors with a small parabola profile or flat reflective strips mounted on linear solar trackers. The mirrors or reflectors direct solar irradiation to a secondary overhead mirror that concentrates and focuses the energy onto an absorber tube. The tube contains a circulating working fluid that can reach temperatures between 60 and 250 °C [23]. The secondary overhead mirror not only reduces unavoidable optical alignment inaccuracies, but it also improves the concentration ratio of the collector. Typically, FLCs have concentration ratios ranging from 10 to 40, while orientation of the collector is achieved by single-axis tracking of the Sun’s position during the day [14]. Parabolic trough collectors (PTC) and cylindrical trough collectors (CTC) have been extensively investigated and some designs have achieved concentration ratios between 15 and 45 [25]. The trough is designed around parabolic or cylindrical mirrors that reflect and concentrate solar irradiation onto an absorber tube. The tube, which contains a working fluid, is located at the focal line of the concentrator. The absorber tube usually consists of a black or selectively coated metal tube that is enclosed within a glass tube. Typically, troughs are mounted on solar trackers that use single-axis sun-tracking systems to follow the sun’s daily motion from east to west. Both PTCs and CTSs operate at temperatures ranging from 50 to 400 °C [24]. Parabolic dish reflectors (PDR) are usually arranged to form an array, with all of the parabolic shaped mirrors directed towards a common focal point. Due to the changing position of the sun during the day, dishes are fitted with two-axis sun tracking systems that maintains the focus of the solar irradiation on the receiver. PDRs can achieve concentration ratios between 100 and 1000, and can reach temperatures of 1500 °C. At the receiver, solar energy is converted to thermal energy, which is then transferred to a suitable circulating working fluid [26]. Because of the high concentration ratios and temperatures, the working fluid can deliver large quantities of thermal energy to drive electrical power generation equipment [33]. Heliostat field collectors (HFC) are mirrors that are arranged to form an array. The mirrors are driven by two-axis sun tracking equipment that enables the mirrors to reflect and focus solar energy towards a common central receiver tower [27]. Because of the large solar energy flux delivered to the receiver, concentration ratios can reach 1500 and temperatures of 2000 °C can be achieved. HFC facilities are equipped with steam generation equipment that can produce high pressure and high temperature steam. The steam is then used to drive high-speed steam turbines, which turn generators to produce electricity [34]. A selection of concentrating solar collectors is presented in Figure 1. 

## 3. Direct Solar Absorption Collectors and the Use of Nanofluids to Improve Efficiency

### 3.1. Direct Solar Absorption Collectors

The problem with non-concentrating solar collectors is there low thermal energy conversion, which prevents their use in high-temperature applications. Collector performance studies have shown they suffer from low incident solar energy capture, inefficient heat transfer, collector heat losses, and low absorption properties of the working fluid [32,35]. Furthermore, absorbers are coated with optically selective coating such as chromium black to improve absorbance, but many of these coating materials are toxic and pose an environmental hazard [36]. 

In contrast, concentrating solar thermal collectors can achieve the high thermal energy transfers needed to produce high-temperatures and pressures needed for electricity generation. However, these collector configurations are expensive and are only economically competitive on a large scale. However, during the late 1970s, considerable interest into creating alternative collector designs to overcome the shortcomings of non-concentrating collectors resulted in the development of the direct solar absorption collector. The objective of this new design was to produce large energy transfers using conventional solar collector configurations [37]. However, several design factors limit the efficiency of traditional solar collectors. These factors include the type and number of transparent covers, surface properties of the collector, size of the collector, and the materials used to manufacture the collector. Accordingly, all these factors must be taken into account when designing a solar collector. In particular, the convective heat flow rate *Q* is an important factor in determining the efficient operation of a solar collector and can be evaluated using Equation (1),

(1) Q=hA∆T

**Figure 2:**
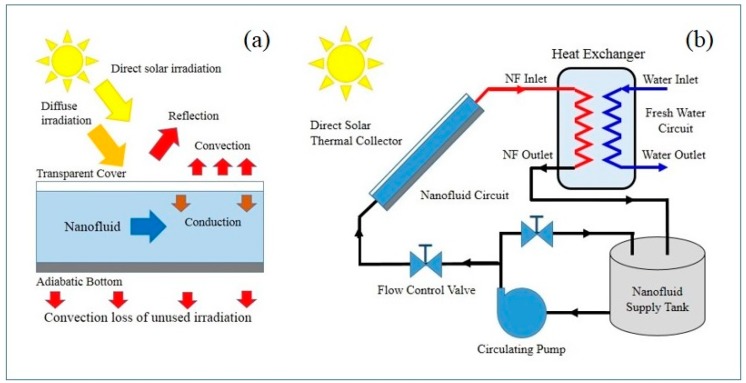
Direct solar thermal absorption collector schematics: (**a**) representative collector showing sources of irradiation and major sources of heat loss; and (**b**) simplified closed loop system that transfer’s heat from the nanofluid circuit to the water circuit via a heat exchanger.

In Equation (1) *h* is the heat transfer coefficient (Wm^−2^K^−1^), A is the surface area of the heat transfer region (m^2^), and Δ*T* is the temperature difference resulting from the heat flow (K) [38]. Inspecting Equation (1) reveals that one or all of the parameters can manipulated to improve the heat flow performance. In the case of a solar collector, increasing *A* is not feasible because the collector size becomes large and heavy. While increasing Δ*T* requires improving the solar concentration ratio, which may not be possible due to collector design. Therefore, to overcome these limitations direct solar absorption collectors were designed to use the working fluid as both solar absorber and heat transfer medium. Thus, thermal properties of the working fluid ultimately determine the performance of the collector. Figure 2a presents a schematic of a representative direct solar thermal absorption collector (with a nanofluid as the working fluid) and shows the source of irradiation and the various heat losses the collector experiences. While Figure 2b presents a typical direct solar thermal absorption design with a closed-loop nanofluid circuit and separate fresh water circuit.

### 3.2. Improving the Thermal Properties of the Working Fluid 

Studies have shown increasing the heat transfer coefficient *h* can significantly improve working fluid performance. However, current working fluids such as water, ethylene glycol, water/ethylene glycol mixtures and oils have thermal conductivity values lower than metals and glasses that go to make up collectors as seen in Table 2. Since most solids have superior thermal conductivity values compared to working fluids, an effective method of enhancing the fluids thermal conductivity is to add small amounts of solid particles. Recent studies have confirmed suspending small particles in a fluid can improve its heat transfer performance [39,40,41,42,43,44,45]. In particular, the addition of small micrometer-scale particles (solid phase) to a working fluid (fluid phase) can improve its thermal conductivity and heat transfer coefficient [46,47]. Unfortunately, two-phase fluids containing micrometer-scale particles have a number of operational problems. These problems include: (1) particle sedimentation that results in reduced heat transfer rates; (2) high erosion rates caused by circulating particles; (3) particles tend to accumulate and block narrow flow channels; (4) increased flow resistance and larger pressure drops, and (5) improved thermal properties are only achieved at large particle concentrations, which in turn increases the magnitude of the above-mentioned problems [48,49]. Because of the problems, the use of micrometer-scale particles in working has not gained widespread acceptance. However, the use of nanotechnology-based techniques has revitalized interest into developing two-phase fluids. The ability to manufacture nanometer-scale materials with physiochemical, electronic and optical properties that are different from their bulk equivalents has created the opportunity to develop a new class of fluids known as nanofluids. Nanofluids are colloidal suspensions containing dispersed nanometer-scale particles. Nanomaterials used in nanofluids to date include metals, non-metals and carbons. These nanomaterials also come in a wide variety of morphologies that include particles, fibers and tubes [50,51,52]. Nanofluids with low viscosities, high thermal conductivities and superior photo-thermal properties are highly desirable for use direct solar absorption collectors [53,54,55,56]. However, issues such as nanofluid stability over time need also to be considered [57,58]. In spite of stability concerns, several studies have reported the use of nanofluids and their ability to improve the long-term performance of direct solar energy collectors [56,59,60,61,62].

## 4. Nanofluids for Use in Direct Solar Absorption Collectors

In recent years, nanotechnology-based techniques have produced nanometer-scale particles smaller than 100 nm. Nanoparticles can be used to form a stable suspension and improve the thermal properties of the base fluid. Studies have shown the addition of small concentrations of metal or metal oxide nanoparticles to a fluid can improve its thermal conductivity value [63,64,65]. Studies have also shown a number of factors can directly influence the thermal properties of a nanofluid. These factors include: (1) concentration of nanoparticle or volume fraction; (2) particle size and morphology of the suspended nanoparticles; (3) temperature of the nanofluid; and (4) distribution of the dispersed nanoparticles [66,67,68,69]. Importantly from a direct solar absorption collector point of view, the addition of nanoparticles can change the optical properties of the fluid from being mostly transparent to highly absorbing over the solar spectrum [17,18,70,71]. Because of the importance of producing nanofluids with long-term stability, the following sections discuss the synthesis and stabilization techniques used to manufacture nanofluids.

### 4.1. Synthesis and Stabilization of Nanofluids

A nanofluid can be produced by mixing a nanoparticle-based powder in a suitable base fluid. The mixture then undergoes sonication to break up any particle agglomerations formed during mixing to create a well-dispersed nanoparticle suspension. After sonication, a stabilizing agent is added to the suspension to prevent further nanoparticle clustering. The addition of a stabilizing agent is important, since many nanometer-scale materials are hydrophobic and will agglomerate to form particle clusters that precipitate out of solution. Furthermore, nanoparticle surface reactivity means a reaction with both fluid and stabilizing agent will take place to produce an interfacial layer. The resulting interfacial layer will directly influence the functionality of the nanofluid. Furthermore, the nanofluid will only remain stable if columbic repulsion of the nanoparticles is greater than the attracting van der Waals forces. Otherwise the nanoparticles will cluster and form flocculates and aggregates [82,83]. Thus, nanoparticle clustering must be avoided, since the solid phase must be dispersed for the nanofluid to retain its unique properties and functionality. On the other hand, stabilizing agents can change the chemical nature of the nanofluid and influence its optical, thermal and hydrodynamic properties [57]. Therefore, a balance must be achieved between all constituents without compromising nanofluid performance. 

### 4.2. Synthesis Techniques

Due to the high demand for nanoparticle-based materials, two manufacturing approaches have developed over the years. The first is the top-down approach and involves reducing the size of bulk materials by physical or chemical processes. The final particle size, shape and surface structure are all dependent on the processing technique used. Furthermore, during the top-down approach surface imperfections are produced and ultimately influence the physicochemical properties of the manufactured nanoparticles. The second is the bottom-up approach and involves the assembly of individual atoms, molecules and smaller particles. Both approaches use a wide variety of physical and chemical manufacturing processes, which have evolved to produce nanoparticles with different sizes, shapes and compositions. For example, a typical top-down physical process involves grinding bulk materials down to the desired particle size. A more complex physical method is evaporating bulk metal precursors in an inert-gas atmosphere, which is then followed by vapor condensation. During vapor condensation, nanoparticle sizes and the particle size distribution are controlled by regulating the condensation conditions. Other techniques that use metal evaporation include arc plasma, laser ablation, lithography, high-energy irradiation and resistive heating [84,85,86,87]. Alternatively, chemical processing techniques such as chemical reduction, electrochemistry and photochemical reduction are used to produce nanoparticles [88,89,90,91]. Wet chemical synthesis is a popular route that reduces metal salt solutions in the presence of a stabilizing agent. Particle size, size distribution, shape and physicochemical properties are all influenced by process parameters. These parameters include initial reagent concentrations, temperature and reaction mixture pH. Moreover, during synthesis there are interactions taking place between precursor ions, reducing agents and the adsorption kinetics of the stabilizing agent. The competing parameters ultimately dictate the properties of the manufactured nanoparticles [92,93]. Because of these dynamic and competing parameters, current research are focused on developing a complete understanding of the formation mechanisms and how to effective control them to produce nanoparticles with specific physicochemical properties [94].

Nanofluids can be thought of as composite materials consisting of a discrete solid phase (nanoparticles) and a continuous liquid phase. However, nanoparticles have large surface areas, high surface reactivity’s and are constantly colliding (Brownian motion) to form particle clusters that readily precipitate out of solution. Particle clustering, precipitation and sedimentation are serious drawbacks. However, recent research has focused on overcoming these drawbacks. Studies have looked at using a one-step method to produce nanoparticles within the working fluid. For example, Eastman et al. developed a one-step evaporation/condensation method to produce copper (Cu)/ethylene glycol nanofluids. The fluids contained small concentrations of Cu nanoparticles (smaller than 10 nm) that equated to a dispersed volume fraction of 0.3% [67]. However, the shortcoming of the one-step method is the presence of residual reactants not used in nanoparticle synthesis. The unused reactants become contaminants and are difficult to remove. The one-step method is in direct contrast to the more traditional two-step approaches. In two-step approaches, the first step produces the nanoparticles, and the second disperses the nanoparticles in the fluid. The advantages of the two-step approach are cost effectiveness and the ability to produce large volumes of nanofluids. This is because many nanoparticle-based powders are commercially available [95,96,97]. 

### 4.3. Nanofluid Stabilization Methods and Stability Evaluation Methods

Nanofluid stability is very important, and in spite of nanoparticles having extremely small sizes and relatively high kinetic energies resulting from Brownian motion, they do not remain in suspension. With time, nanoparticles settle out of solution under the influence of gravity. While in solution nanoparticle motions are the result of interactions involving van der Waals forces, electrical double layer action and steric action. Balancing the various competing interactions leads to nanoparticle dispersion and prevents clustering and sedimentation. From a practical point of view, clustering and sedimentation cause two problems. The first results from the loss of photo-thermal and thermal properties of the nanofluid. The second results from the build-up of sediments in the solar collector. The circulating sediments cause tube wall abrasion and the accumulating sediments reduce flow. Therefore, keeping nanoparticles dispersed is critical for the stability and performance of the nanofluid. Section 4.3.1 discusses several physical and chemical methods used to keep nanoparticles in suspension. While Section 4.3.2 reviews several evaluation methods for determining nanofluid stability.

#### 4.3.1. Methods to Enhance Nanofluid Stability

##### Physical Methods 

Physical methods used to promote nanofluid stability include mechanical agitation, stirring and ultrasonic vibration [98]. Mechanical stirring techniques have been used by several researchers, where the working fluid was distilled water and titanium oxide (TiO_2_) and aluminum oxide (Al_2_O_3_) were the respective solid phases in the nanofluids [99,100]. While several articles have reported the use of ultrasonic vibration to disperse and suspend a variety of nanoparticles in different base fluids. For example, Duan et al. have investigated the influence of sonication time on particle size and its influence on viscosity for Al_2_O_3_—water nanofluids [101]. While Eastman et al. [102], Lee et al. [66], and Wang et al. [100] have used two-step synthesis methods to produce Al_2_O_3_ nanofluids under the influence sonication and stirring to reduce particle clustering. A study by Hong et al. found increasing sonication times lead to less particle clustering during the synthesis of iron (Fe)/ethylene glycol nanofluids [103]. Whereas, the influence of sonication time and power on long-term stability of TiO_2_—water nanofluids were studied by Lotfizadeh Dehkordi et al. Their study found increasing sonication time and power during synthesis reduced particle clustering [104].

##### Effect of Surfactants 

Physical dispersion methods are unsuitable for nanofluids used in direct solar absorption collectors. Instead, chemical methods are used. Steric stabilization is a chemical method that introduces small amounts of surfactant into the nanofluid to modify the surface properties of the nanoparticles. Surfactants are amphiphilic compounds that contain a hydrophobic tail and a hydrophilic polar head group [105]. The hydrophobic tails attach to the naturally occurring hydrophobic nanoparticles, while the hydrophilic polar heads radiate out to form a hydrophilic outer layer that interacts with the surrounding polar fluid (i.e., water). Thus, surfactants improve nanoparticle wettability by reducing surface tension and promoting greater fluid continuity. Therefore, selecting the correct surfactant is a very important factor that must be considered when producing a stable nanofluid. Surfactants come in four classes. The classes are based on head composition and include amphoteric, cationic, anionic, and non-ionic [106]. As a rule, nanofluids composed of a polar solvent should use a water-soluble surfactant. While nanofluids composed of non-polar fluids (i.e., oil) should use an oil-soluble surfactant. However, in spite of the wide range of commercially available surfactants, several problems have been reported in the literature [107]. For example, several studies have reported increasing surfactant concentrations can increase nanofluid viscosity [108,109]. Further problems include foam generation, contamination and lower heat transfer properties. In particular, irreversible surfactant deterioration has been reported for temperatures above 60 °C. The resulting deterioration produces instability, particle aggregation and sedimentation [110].

##### Surface Functionalization of Nanoparticles 

Surfactants can improve stability, but problems such as contamination, foaming and loss of thermal properties can reduce nanofluid performance [98,111,112]. Surface functionalization is a surfactant-free approach that can deliver long-term nanofluid stability without the problems normally associated with surfactants. Several approaches are reported in the literature. For example, Joni et al. have used nitrogen doping during bead-milling to produce titanium dioxide (TiO_2_) nanoparticles [113]. While Kulkarni et al. have examined the functionalization of copper oxide (CuO) nanoparticles [114] and Yang et al. have grafted silanes directly onto silica (SiO_2_) nanoparticles [58]. Similar studies have also looked at grafting polymeric or functional moieties onto different types of nanoparticles for use in nanofluids and nanocomposite materials [115,116]. For example, Tang et al. have used polymethacrylic acid (PMAA) to functionalize the surface of Zinc oxide (ZnO) nanoparticles. In their study, they found the hydroxyl groups present on nanoparticle surfaces interacted with the carboxyl groups of the PMAA. The resulting interactions formed poly (zinc methacrylate) complexes, which promoted greater dispersion and prevented particle agglomeration [117]. In recent years, carbon materials (graphite, graphene, carbon black, carbon nanotubes) and a variety of fluids (water, alcohols, glycols, oils, and their mixtures) have been investigated for the manufacture of nanofluids [118,119,120]. Carbon materials have a hydrophobic character, which makes them prone to aggregation and precipitation in water. In terms of surface functionalization, Hwang et al. have used mechanical/chemical reaction techniques to deposit hydrophilic functional groups onto carbon nanotubes to prevent aggregation [57]. While Chen et al. have used a surfactant free technique to deposit hydroxyl groups onto double and single walled carbon nanotubes. The surface hydroxyl groups were found to dispersed both double and single walled carbon nanotubes in their respective fluids and promote nanofluid stability [121]. Alternatively, Qu et al. have used a plasma-base technique using gas mixtures (methane and oxygen) to deposit polar groups on diamond nanoparticles. The polar groups were found to improve the dispersion properties of diamond nanoparticles in water [122]. 

##### pH Control of Nanofluid Stability

Controlling the surface charge on nanoparticles by regulating fluid pH is a technique that can increase nanofluid stability. Creating a high surface charge produces an electrical double-layer around the nanoparticle, which results in strong repulsive Coulombic forces that promote particle dispersion [98]. pH variation effects on dispersion stability and other properties of the resulting nanofluid have been studied by several researchers over the last decade. For example, Xian-Ju et al. have studied the effect of pH on dispersion stability and viscosity for alumina (Al_2_O_3_) and Cu based aqueous nanofluids. In their studies, sodium dodecyl benzene sulfonate (SDBS) was used as the surfactant, while the addition of either hydrochloric acid (HCl) or sodium hydroxide (NaOH) were used to control the pH value of the respective nanofluids. The studies found viscosity of alumina-based fluids were generally higher than Cu-based fluids for the same weight fraction and pH value. Dispersed and stable nanofluids were produced for particular pH ranges (alumina-based: between 7.5 and 8.9, Cu-based: 7.6). However, when the pH value was below 7 both nanofluid types experienced instability that resulted in agglomeration and rapid sedimentation [123]. A study by Yang et al. also examined the rheological behavior of TiO_2_ loaded nanofluids when the pH value was varied. Their study investigated the effect of varying the pH value between 1.2 and 9.1, and its effect on viscosity for a 54.5 wt % (24 vol %) TiO_2_, (mean particle size of 0.24 µm) nanofluid. The highest viscosities were recorded for pH values between 5 and 6.5, while lower viscosities were observed for nanofluids with a pH value of 9. However, their study did not report on the long-term stability of the nanofluids [124]. Similarly, a study by Jia-Fei et al. found the pH value influenced the viscosity and stability of silicon dioxide (SO_2_) based nanofluids with particle sizes around 20 nm. Variations in pH between 5 and 7 produced significant increases in viscosity. While pH values less than 5 produced lower viscosities [125]. Other studies have also shown pH values that vary from the nanoparticles isoelectric point (IEP), the colloidal suspension (nanofluid) becomes more stable [126,127]. For example, acid washing of carbon nanotubes can improve their dispersion in water-based nanofluids [128,129]. While studies have also shown the variation of pH can change nanofluid thermal properties [110,123,130]. For example, a study by Amrollahia et al. revealed pH was an important factor in determining both nanofluid stability and thermal conductivity [131]. Furthermore, Yousefi et al. found variation of pH with respect to the isoelectric point could improve the performance a multi-walled carbon nanotube-based aqueous nanofluids in solar collectors [132].

#### 4.3.2. Evaluation Methods for Determining Nanofluid Stability

Long-term nanofluid stability is important, but nanoparticles forming the solid phase are in constant Brownian motion and sooner or later interact with neighboring particles. During these interactions, strong attractive forces produce particle clustering that results in aggregation. The agglomerations progressively get larger and under the influence of gravity begin to settle making nanofluids unstable. Besides blocking flow channels in collectors, the sedimentation process also reduces the thermal performance of nanofluids. Therefore, having manufactured a nanofluid it is important to evaluate its long-term stability. The following sections discuss various techniques used to evaluate nanofluid stability.

##### Zeta Potential Measurement

The stability of a nanofluid can be determined by measuring the electrical potential between the dispersion medium and the stationary fluid layer attached to the particle. The electrical potential is known as the zeta potential and indicates the degree of repulsion between charged particles dispersed in the fluid. A high zeta potential indicates strong columbic repulsion forces between the dispersed particles and smaller attractive van der Waals forces. Nanofluids with high zeta potentials (negative or positive) are considered to be electrically stable. While nanofluids with low potentials will undergo nanoparticle clustering and sedimentation. Nanofluids with zeta potentials between 40 and 60 mV are considered stable. While nanofluids with zeta potentials greater than 60 mV have excellent stability [106]. Moreover, because of straightforwardness of the technique, zeta potential measurements are routinely used to determine nanofluid stability [133,134,135].

##### Sedimentation and Centrifugation Methods

Studying sedimentation behavior is an important method for evaluating long-term nanofluid stability for a particular application. Sedimentation is a straightforward method, but is time consuming since it relies on gravity to cause particle settling in batch columns. Because of the time involved, photography is used to capture a series of images taken over hours, days and even months [136,137]. Nanofluids are considered stable if no sedimentation takes place over time. Several researchers have used sedimentation as a method of determining long-term stability for a wide variety of nanofluids including Cu-H_2_O, TiO_2_-H_2_O and kaolinite (Al_2_O_3_·2SiO_2_·2H_2_O) in aqueous media [138,139,140]. Moreover, since sedimentation takes a long time some researchers have also used centrifugation to speed up the process. During centrifugation, the centrifugal force is many times greater than normal gravity and sedimentation takes place more rapidly [141,142]. For example, Singh et al. found silver (Ag) nanofluids synthesized by the reduction of silver nitrate (AgNO_3_) in ethanol with polyvinyl pyrrolidone (PVP) acting as the stabilizing agent were stable using conventional sedimentation. Furthermore, when subjected to centrifugation for more than 10 h at 3000 rpm, no sedimentation was detected. Thus, confirming the Ag nanofluids were very stable [143].

##### UV–Visible Spectroscopy Analysis

UV–Vis spectral analysis has been used by several researchers to determine nanofluid stability [49,138,144]. The technique is used when the solid phase of the nanofluid has absorption bands between wavelengths of 190 and 1100 nm. Nanofluid stability is determined by monitoring the absorption characteristics, which are equivalent to particle concentration in the fluid over time. The technique has been used on a wide range of nanofluids that include Ag-H_2_O, gold (Au)-H_2_O, TiO_2_-H_2_O, and a variety of carbons materials (carbon nanotubes, graphene) [104,143,145,146]. However, the technique becomes difficult to use for nanofluids with high particle concentrations and have dark colors. The dark color also makes it difficult to see any sedimentation products [98].

##### Dynamic Light Scattering (DLS) Method

Dynamic Light Scattering (DLS) is a popular technique used to measure particle sizes down to 1 nm. The technique uses a laser to illuminate dispersed nanoparticles in a fluid sample. The nanoparticles rapidly move throughout the sample due to Brownian motion. A photon detector records the resulting scattered light fluctuations, converts the data to particle velocity and then calculates particle size and particle size distribution from the velocity data. Thus, the DLS technique is used to monitor nanofluid stability over time by measuring the variation in particle size. Stable nanofluids will have a constant mean particle size over time, while unstable nanofluids will have increasing particle sizes with time. Thus, DLS technique is used to monitor the long-term stability of both water-based and oil-based nanofluids [147,148].

## 5. Types of Nanofluids

### 5.1. Pure Metals, Metal Oxides and Carbide Based Nanofluids

Improvement of the thermal properties of thermo-fluid by the incorporation of nanoparticles was initially proposed by Choi in 1995 [149]. Since then there has been considerable research into developing nanofluids with unique thermo-physical properties such as thermal conductivity, thermal diffusivity and viscosity [17,49,59,66,150]. Studies have revealed the thermo-physical properties of several fluids can be improved by the addition of small concentrations of nanoparticles. Types of nanoparticles investigated include pure metals (Au, Ag, Cu, Al, and Fe), metal oxides (Al_2_O_3_, CuO, Fe_3_O_4_, SiO_2_, TiO_2_, and ZnO), Carbides (SiC, TiC) and a variety of carbon materials (diamond, graphite, single/multi wall carbon nanotubes). Many of these nanoparticle types have been incorporated into fluids such as water, water/ethylene glycol, ethylene glycol and oils. In terms of pure metals, both Au and Ag nanoparticles have been studied because of their unusual optical, electronic and chemical properties, which are size and shape dependent [151,152,153]. Both Au and Ag have high thermally conductivities and their addition to fluids for heat transfer applications would be interesting, but very few studies have examined their use in solar collectors. However, a recent study by Filho et al. revealed Ag nanoparticle-based nanofluids had good photo-thermal conversion properties for particle concentrations of 1.62, 3.25 and 6.5 ppm. Their study also found the specific absorption rate (SAR) was only discernable during initial heating, and at higher particle concentrations there was a significant reduction in performance. The reduced performance was the result of factors such as particle clustering near the fluid surface, which in turn reduced sunlight entering the nanofluid [154]. While a recent study by Chen et al., investigated the photo-thermal conversion efficiency of Au-based nanofluids. In their study, Au nanoparticles of varying sizes (25 nm, 33 nm, and 40 nm) were dispersed in water-based nanofluids. The presence of Au nanoparticles was found to enhance solar absorption with respect to pure water for small particle concentrations. Trials in cube shaped and flat shaped direct solar thermal absorption collectors revealed photo-thermal conversion efficiencies of 19.9% and 21.3% respectively [155]. However, because of the cost of noble metals, several researchers have investigated less noble metals and alternative materials. For example, Eastman et al. have used combinations of Cu nanoparticles and ethylene glycol to produce nanofluids. For a nanofluid with a dispersed Cu nanoparticle volume of 0.3% there was a 40% increase in the fluids thermal conductivity [67]. While in an earlier study using water-based nanofluids, Eastman et al. reported a volume fraction of CuO nanoparticles (5 vol %) could produce a 60% increase in thermal conductivity. Similarly, they also found thermal conductivity enhancements of around 40% for aqueous nanofluids containing small volumes of Al_2_O_3_ nanoparticles (5 vol %) [102]. However, not all studies give the expected thermal conductivity enhancement. For instance, Hong et al. reported thermal conductivity values higher for Fe nanoparticle-based nanofluids compared to those of Cu nanoparticle-based nanofluids for the same volume fractions [156]. A selection of nanofluids containing small concentrations of metal and metal oxide nanoparticles are shown in Table 3 and presents the respective thermal conductivity enhancements reported in the literature. 

In terms of direct absorption solar collectors, Tyagi et al. using water-based nanofluids with small concentrations of aluminum (Al) nanoparticles investigated their performance in flat-plate collectors. Their study found the presence of nanoparticles increased the absorption of incident radiation and improved the efficiency of the collectors by 10% compared to collectors using pure water [16]. In a similar study, Saidur et al. found even a small (1%) volume fraction of Al nanoparticles in water-based nanofluids could improve solar absorption [97]. In terms of oil-based nanofluids, Sokhansefat et al. has numerically studied the heat enhancement of an Al_2_O_3_—synthetic oil based nanofluids when used in parabolic trough/tube collectors. Al_2_O_3_ nanoparticle volume fractions ranging from 1% to 5% were examined over a number of temperatures ranging from 300 to 500 °K. The resulting improvements in heat transfer coefficients over this temperature range were between 5 and 15% compared to collectors using pure synthetic oil [157]. Similarly, Lee et al. have measured the effective thermal conductivities and viscosities of water-based nanofluids containing low-volume fractions of Al_2_O_3_ nanoparticles (0.01–0.3%). The results of their study reveal a near linear increase in thermal conductivity with increasing volume fraction. While the measured viscosity values showed a nonlinear trend over the entire range of volume fractions [158]. Whereas Luo et al. has used numerical modelling to evaluate the radiative transport equations, conduction equations and convection heat transfer equations for several particulate oil-based nanofluids employed in direct solar thermal absorption collectors. To evaluate their modelling, they prepared a selection of nanofluids and used a solar radiation simulator to investigate the performance of each nanofluid. The solid phases used in the respective nanofluids included Cu, Ag, Al_2_O_3_, SiO_2_, TiO_2_, graphite nanoparticles, and carbon nanotubes. All nanofluids used Texatherm oil as the fluid phase. The results of the experimental studies confirmed their modelling predictions. The studies also revealed improved outlet temperatures and collector efficiencies for all nanofluids except those with a TiO_2_ solid phase [45]. While studies carried out by Murshed et al. have shown water-based nanofluids with TiO_2 _nanoparticles have increasing thermal conductivities when the particle volume fraction increases [159]. This study also highlighted the importance of using the appropriate fluid for a specific solid phase to gain the most desirable thermal conductivity enhancements. For further information regarding the performance of nanofluids and their application in solar energy systems, the authors would like to recommend the following three recent reviews by Bozorgan & Shafahi [160], Kasaeian et al. [41] and Reddy et al. [161].

### 5.2. Carbon Nanomaterial Based Nanofluids

#### 5.2.1. Carbon Nanomaterials 

**Figure 3:**
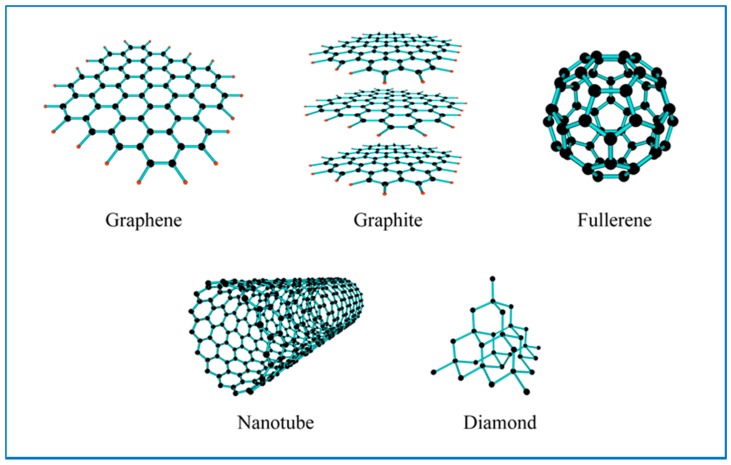
A selection of carbon allotropes that have been examined for use in nanofluids [164,169,175,177].

Carbon is one of the most abundant elements in the Earths biosphere. Nature has used this element, coupled predominantly with oxygen and hydrogen to form a diverse range of organic compounds. Furthermore, carbon atoms can bond with themselves in a number of different ways to form a variety of carbon materials or allotropes of carbon. Each of these allotropes has differences in their respective material properties. Typical carbon allotropes include amorphous carbon, diamond and graphite. Carbon allotropes can also have a variety of structures and morphologies such as crystalline (i.e., diamond, three dimensional, 3D), graphite sheets (2D) and carbon nanotubes (1D). Moreover, the discovery of Buckminster-fullerenes, or Bucky balls by Kroto et al. in the mid-1980s, stimulated the search for new forms of carbonaceous materials [162]. Carbon nanomaterials are of particular interest, since they are black in color, which makes them ideal for solar absorption applications. In addition, their very high thermal conductivity also makes them ideal additives for nanofluids [163,164,165]. For example, the thermal conductivity of carbon nanotubes (2000 to 6000 Wm^−1^K^−1^) are an order of magnitude higher than metals like Au (315 Wm^−1^K^−1^) and Cu (398 Wm^−1^K^−1^), and metal oxides such as Al_2_O_3_ (40 Wm^−1^K^−1^) and CuO (77 Wm^−1^K^−1^) as seen in Table 2. New forms of carbon nanomaterials include: (1) single and multi-wall carbon nanotubes [166,167]; (2) nanoballs [168]; (3) fullerenes C70, C76 [169], C84 [170], C60 in a crystalline form, nanocones [171], nanohorns [172], nanofilaments [173], nanocapsules [174]; (4) graphite nanoparticles [175]; (5) graphene and graphene oxide [176,177]; (6) carbon black [178,179], and (7) carbon nanospheres (CNS) [180,181]. Figure 3 and Table 3 presents a selection of carbon allotropes that are currently being investigated as potential solid additives for carbon-based nanofluids. The following sections discuss the incorporation of carbon nanomaterials into fluids to improve their thermal performance in direct solar thermal absorption collectors.

#### 5.2.2. Carbon-Based Nanofluids for Direct Solar Absorption Collectors 

Several studies have revealed the addition of small quantities of carbon nanomaterials to a fluid (i.e., water) can improve its thermal properties [55,79,121]. Therefore, selecting the appropriate carbon nanomaterial and fluid is important for creating nanofluids with enhanced thermal properties. Studies have shown improved fluid thermal conductivity can be achieved by the addition of small quantities of carbon nanomaterials with high thermal conductivities. The following sections discuss the various carbon allotrope additives used to improve the thermal performance of various types of fluids. 

##### Carbon Nanotube-Based Nanofluids 

Carbon nanotubes (CNTs) are one-dimensional carbon-based cylinders with high aspect ratios. The cylindrical structure is composed of either single walled (SWCNTs) [182,183] or multi-walled (MWCNTs) [130,184,185,186] configurations. CNTs synthesis was first reported by Iijima in 1991 and since then the unique thermo-physical properties of these materials have been extensively studied [166]. An early study by Choi et al. found the addition of small quantities of MWCNTs (1 vol %) dispersed in poly (alpha-olefin) oil could improve the oils thermal conductivity by 160% [79]. Similar studies by Natarajan and Sathish have also shown thermal conductivity values of several fluids used in solar heaters can be improved by the addition of small concentrations of CNTs [187]. Moreover, a recent study examining optical properties, thermal conductivities and dispersion stability of several water-based nanofluids containing small concentrations of CNTs by Karami et al., found improvement in these properties when compared to pure water. Their study used a two-step method, which began by treating the CNTs with carboxylate functional groups. In the second step, functionalized CNTs (150 ppm) were added to the fluid to produce a 32% improvement in thermal conductivity. The presence of the carboxylate functional groups also promoted dispersion stability. Thus, making CNT/water-based nanofluids a viable candidate for low-temperature direct absorption solar collectors [188]. While a recent study by Lee and Jang to determine the extinction coefficient for MWCNTs disperse in water-based nanofluids, found that only a small volume fraction (0.5 × 10^−3^ vol %) was needed to completely absorb all incident solar irradiation at a wavelength of 632.8 nm. The study also undertook comparative studies between the MWCNT-based nanofluids, graphite-based nanofluids and single-walled carbon nano-horn (SWCNH)-based nanofluids previously reported in the literature. The results of the comparison studies revealed for a small volume fraction range (0.5 × 10^−3^ to 0.5 × 10^−2^ vol %) both MWCNT and SWCNH nanofluid extinction coefficients tended to increase linearly. While the graphite-based nanofluid extinction coefficients were nonlinear over the same volume fraction range [189]. The role of surfactants in stabilizing CNT-based nanofluids have been investigated by several researchers. For instance, Thierry Maré et al. have reported using sodiumdodecylbenzene sulfonate (SDS) to stabilize water-based CNT nanofluids for use in heat exchangers and energy systems [190]. Whereas, Fadhillahanafi et al., have reportedly used PVP as a stabilizing agent in water-based MWCNT nanofluids. Their study revealed thermal conductivity values for nanofluids with PVP were higher than those without the surfactant. In particular, nanofluids with small mass fractions (~0.5 wt %) and small concentrations of PVP (~0.01 wt %) produced thermal conductivity enhancements of around 22.2% [129].

##### Nano-Diamond Nanofluids 

In recent years, several researchers have appraised the thermal conductivity properties of nano-diamond nanofluids [191,192]. Studies by Ghazvini et al. investigated nanofluids composed of water/engine oil mixtures and small quantities of nano-diamonds (ND). They found the addition of small mass fractions (~1.0 wt %) could produce thermal conductivity enhancements of around 25% [193]. While studies by Ma et al. found the addition of small quantities of NDs (0.01 vol %) to water-based nanofluids increased the pure waters thermal conductivity from 0.581 W m^−1^K^−1^ to 1.003 W m^−1^K^−1^ [194]. Similarly, Yu et al. have reported adding small volume fractions (~1.0 vol %) of NDs to ethylene glycol to produce thermal conductivity enhancements of around 17.23% [195]. While Taha-Tijerina et al. have added small amounts of NDs (0.1 wt %) to mineral oil-based nanofluids to deliver thermal conductivity enhancements of around 70% [192]. On the other hand, Assael et al. found small quantities (~1.0 wt %) of poorly dispersed NDs in aqueous mixtures containing a surfactant (SDS, 45.0 wt %) only produced thermal conductivity enhancements of 2% [196].

##### Graphite and Graphene Nanofluids

Several researchers have investigated the change in thermal properties of fluids after the addition of small quantities of graphite or graphene [175,197,198,199]. Ladjevardi et al. have used numerical modelling and experimental studies to examine the influence of particle size and volume fractions on the performance of graphite nanofluids in direct absorption solar collectors. Their study found small volume fractions (~0.25 × 10^−5^ vol %) of nanometer-scale graphite in water absorbed 50% of all incident solar irradiation, while pure water absorbed only 27% [175]. Studies by Taylor et al. have also examined the influence of adding small quantities of graphite nanoparticles to water-based nanofluids designed for solar collectors. Their studies demonstrated that it was possible to achieve collector performance improvements of around 10% [200]. Whereas, Eswaraiah et al., have examined the mechanical properties resulting from the addition of graphene to engine oil-based nanofluids. The study revealed the bearing action of graphene and its high strength enhanced the mechanical properties of the respective nanofluids [201]. Furthermore, Ghozatloo et al. have examined the thermal conductivity enhancement of water-based nanofluids when small amounts of grapheme were added. The study revealed the convective heat transfer coefficient at 38 °C was increased by 35.6% when a small amount of graphene (0.1 wt %) was added to the nanofluid. The study also found the addition of small amounts of graphene (~0.075 wt %) produced thermal conductivity improvements of around 32% [202].

##### Carbon Black and Other Carbon-Based Nanofluids

Literature in the field is limited, but a small number of studies were carried out to investigate the effects produced by the addition of other carbon-based materials to nanofluids. For example, Kirilov et al. have examined properties such as wettability and surface adsorption characteristics of carbon particles treated with heated hydrogen peroxide (H_2_O_2_) solutions. The treated carbon particles were then dispersed in a fluid mixture consisting of water and propylene glycol. The resulting carbon-black nanofluids displayed good photo-thermal properties and produced significant temperature increases over time when exposed to solar irradiation [203]. Carbon-black nanofluids have also been studied by Han et al. to determine their thermal conductivities, optical properties and photo-thermal responses to solar irradiation. Their studies revealed the properties tended to increase with increasing volume fractions of carbon-black in the water-based nanofluids. The studies also confirmed carbon-black nanofluids could be used in solar thermal applications [55]. While in a study by Meng et al., the thermal conductivities of carbon-black loaded ethylene glycol-based nanofluids were found to increase with particle loading and temperature. Their study also confirmed the feasibility of using carbon-black nanofluids in direct solar absorption collectors [204]. Whereas, Poinern et al. have produced carbon nano-spheres (CNS) from the pyrolysis of acetylene gas. The resulting CNSs were then incorporated into water-based nanofluids [181]. Their study found increasing quantities of CNSs in the nanofluids improved their photo-thermal response and confirmed the use of CNS-based nanofluids in direct solar absorption collectors. Alternatively, Karami et al. have examined the enhanced optical properties resulting from the inclusion of small quantities of carbon nano-balls (300 ppm) in nanofluids composed of water and ethylene glycol. The transmittance data collected during their study revealed the water-based nanofluids were superior absorbers [168]. Also using ethylene glycol-based nanofluids, Sani et al. were able to demonstrate long-term stability (greater than 6 months) for fluids containing small quantities of dispersed single-wall carbon nano-horns (SWCNHs) [51].

## 6. Modelling Nanofluid Thermal Conductivity 

The thermal conductivity of fluids used in solar thermal collectors are much lower than solid materials as seen in Table 2. Because solid materials have superior heat transfer properties, small metallic or non-metallic particles are used as additives to enhance the thermal performance of fluids. When nanoparticles are added, the resulting dispersion is known as a nanofluid. However, anomalous property behavior has been reported. For example, measurements have revealed the anomalous behavior tends to improve the fluids thermal conductivity value. Over the years, numerous theoretical models were developed to try and predict the thermal conductivity of fluids containing a small volume of dispersed solid phase of particles. The first model aimed at explaining the thermal conductivity enhancements seen in fluids containing small spherical particles was developed Maxwell [214]. Maxwell's model is designed for small concentrations of small non-interacting spherical particles dispersed in a continuous homogeneous isotropic material. The nanofluids effective thermal conductivity (*k_eff_*) is determined by Equation (2), which incorporates the thermal conductivities of the solid phase (*k_s_*) and fluid (*k_f_*). While the volume fraction is ϕ and β expresses the ratio of thermal conductivities (β = *k_s_*/*k_f_*).

(2) $$\frac{k_{eff}}{k_{f}}=1+\;\frac{3\left(\beta -1\right)\emptyset }{\left(\beta +2\right)-\left(\beta -1\right)\emptyset }$$

However, limitations in the model has resulted in several researchers modifying its structure to take into account factors such as particle size, particle shape, types of fluids, the effect of respective phase thermal conductivities and temperatures [48]. While other studies have suggested incorporating mechanisms such as Brownian motion, a liquid-solid interface layer surrounding the dispersed nanoparticles and particle clustering to explain the thermal conductivity enhancements seen in nanofluids [215]. Thus, modelling nanofluid properties such as thermal conductivity presents a major challenge that involves developing a formal theoretical basis and understanding of the various mechanisms involved to explain the experimental results reported in the literature. A selection of significant models developed by researchers to overcome some of the shortcomings of Maxwell’s model is presented in the following discussion. However, a more detailed mathematically analysis and discussion of thermal conductivity modelling can be found in the literature [216,217,218,219].

Hamilton and Crosser extended Maxwell's equation to take into account the influence of non-spherical particles [220]. The inclusion of an empirical shape factor (*n* = 3/ψ) was designed to take into account the non-spherical nature of particles. The sphericity (ψ) is the ratio between the surface area of a spherical particle and the surface area of a non-spherical particle, with both having equal volumes. Once the shape factor is incorporated, the effective thermal conductivity can be calculated using Equation (3).

(3) $$\frac{k_{eff}}{k_{f}}=1+\;\frac{n\left(\beta -1\right)\emptyset }{\left(\beta +n-1\right)-\left(\beta -1\right)\emptyset }$$

When the ratio of thermal conductivities is less than 100 (β < 100), the shape factor *n* becomes 3 and Equation (3) is equivalent to Maxwell’s equation. However, when the ratio of thermal conductivities is greater than 100 (β > 100), the shape factor is defined by *n* = 3/ψ. Thus, the shape or aspect ratio of a particle directly influences a nanofluids thermal conductivity. For example, Evans et al. studying clustering and interfacial thermal resistances found nanoparticles with high aspect-ratios, instead of spheres, gave larger thermal conductivities for specific particle concentrations [221]. Similarly, Murshed et al. found cylindrical TiO_2_ nanoparticles (10 nm × 40 nm) produced slightly higher thermal conductivities than spherical nanoparticles (15 nm) in water-based fluids [159]. A drawback of Hamilton and Crosser's model, like Maxwell's model, is that it does not take into account the size of the dispersed particles. Importantly, recent studies have shown the high surface area-to-volume ratio of nanoparticles in suspension is an important factor. This is because nanoparticles have more of their constituent atoms located near the surface where they can readily interact with the surrounding fluid [65,66]. In spite of the shortcoming, Equations (2) and (3) give reasonable results for nanofluids composed of dilute nanoparticle concentrations [49]. 

Nanofluid thermal conductivity enhancement can be reduced if the thermal conductivity of the fluid phase increases for a particular nanoparticle type. Also, changes in temperature directly influences Brownian motion, which impacts on nanoparticle behaviors such as particle collisions, thermal interactions and diffusion [222,223,224]. Recent studies have suggested several models to explain the contribution of Brownian motion to thermal conductivity enhancement. However, Brownian motion by itself cannot explain the thermal conductivity improvements seen in nanofluids. The high surface area to volume ratio promotes not only large heat transfers between individual nanoparticles, but also transfers to surrounding fluid particles. Furthermore, as nanoparticles randomly travelling through the fluid they also carry attached fluid molecules along with them creating micro-scale convective effects that promote greater thermal conduction [219,225]. Thus, recent modelling and experimental studies have examined the influence of particle size, fluid type and thermal interfacial resistance between nanoparticles [226,227,228]. Furthermore, several researchers have examined the influence of particle clustering and nano-layer formation resulting from the attachment of fluid molecules to the nanoparticle surface as possible contributing mechanisms to thermal conductivity [229,230]. Consequently, recent studies have investigated the influence of nano-layer thickness and heat transfer between nanoparticle and the surrounding fluid environment [230,231]. Beginning with Maxwell's model, Yu and Choi incorporated δ (ratio of the nano-layer thickness to the original particle radius) to account for the influence of an ordered nano-layer composed of fluid molecules surrounding a dispersed nanoparticle. In addition, they also replaced the solid phase thermal conductivity term (*k_s_*) with a modified nanoparticle thermal conductivity term (*k_se_*) [232]. Thus, the effective thermal conductivity for a nanofluid, incorporating a nano-layer, can be calculated using Equation (4).

(4) $$\frac{k_{eff}}{k_{f}}=\;\frac{k_{se}+2\;k_{f}+2\left(k_{se}-\;k_{f}\right)\;{\left(1+\;\delta \right)}^{3}\;\emptyset }{k_{se}+2k_{f}-\left(k_{se}-k_{f}\right)\;{\left(1+\;\delta \right)}^{3}\;\emptyset }$$

Similar studies have modelled the influence of nano-layering and its outcome on effective thermal conductivity for several nanofluids with varying degrees of success [233,234]. However, studies have also shown the importance of nanoparticle clustering when determining the effective thermal conductivity of nanofluids [235,236]. For example, Feng et al. have proposed a model that incorporates nano-layered nanoparticles forming clusters under the influence of attractive van der Waals forces [237]. During cluster formation, Brownian motion declines due to the increasing cluster masses. At the same time, effective thermal conductivity increases due to increased contact between nanoparticles forming the clusters [238]. Conversely, as larger clusters form and precipitate out of solution, the effective thermal conductivity of the nanofluid decreases [235]. 

From the above discussion, it is clear there is no definitive model that fully explains the anomalous enhancement of thermal conductivity seen in nanofluids. Among the models discussed, few are able to fully explain all the mechanisms involved in thermal conductivity enhancement. Furthermore, no single factor has been clearly identified as the reason for high thermal conductivity enhancements. Instead a combination of factors has been identified as contributors to the overall effective nanofluid thermal conductivity. Factors identified were nanoparticle size, nano-layer thickness, micro-scale convective effects produced by Brownian motion and nanoparticle clustering. Although there have been many studies reporting thermal conductivity enhancement, few studies have reported other property enhancements such as surface tension and viscosity [150]. In particular, viscosity is an important factor in determining the pumping power needed to circulate working fluids around solar collector circuits. Recent studies have confirmed the existence of viscosity enhancements in several nanofluids [239]. However, a recent review article by Mishra et al. has revealed significant variations between experimental measurements and viscosity values predicted by current theoretical modelling [240]. Thus, highlighting the need for further research into developing more effective modelling that can be used to design and improve the performance of direct solar thermal absorption collector systems.

## 7. Challenges Facing the Use of Nanofluids in Direct Solar Thermal Absorption Collectors

Nanofluids offer several attractive and beneficial thermo-physical properties that can improve the performance of direct solar thermal absorption collectors. In particular, thermal conductivity enhancements seen in a number of nanofluids has encouraged many researchers to evaluate their performance in direct solar thermal absorption collectors as seen in Table 4. The results of several studies are presented in Table 4, and confirm the addition of small quantities of nanometer-scale materials can improve the thermo-physical properties of working fluids. In particular, the studies have confirmed the addition of small nanoparticle concentrations can have a significant impact on thermal conductivity enhancement. However, studies have also identified a number of factors that hinder the long-term stability and viability of nanofluids for practical use in solar thermal applications. Factors include nanoparticle clustering, agglomeration and precipitation with time. Furthermore, the addition of surfactants and additives to alleviate these factors will also change the thermo-physical properties of the nanofluid. For example, increasing concentrations of surfactants will increase nanofluid viscosity and produce larger pressure drops throughout the collector system. While the larger pressure drops result in larger demands for pumping power to circulate the working fluid. Furthermore, factors such as changing thermo-physical properties with temperature, particle size variation, changing particle volume fraction and photo-thermal degradation all need further investigation.

## 8. Conclusions

Direct solar thermal absorption collectors offer a clean and viable renewable energy source. Their use can alleviate the detrimental consequences of greenhouse gas emissions, global warming and environmental degradation. Unfortunately, water used in solar thermal collectors can only absorb around 13% of incoming solar irradiation. Because of waters low absorbance, research has focused on using nanofluids with enhanced thermo-physical properties to improve solar thermal collector performance. The review has presented an overview of recent research into nanofluids and their use in direct solar absorption collectors. Important nanofluid parameters such as base fluid properties, types of solid phase, nanoparticle size and concentration, synthesis techniques and nanofluid stabilization methods were discussed. In spite of many advantages, poor long-term nanofluid stability needs to be addressed. Stability related problems include nanoparticle clustering, precipitation and sedimentation. The addition of surfactants and additives can prevent clustering and precipitation, but their use can have a detrimental impact on nanofluid properties. The review has also discussed the thermal conductivity enhancement seen in many nanofluids, and the subsequent performance improvements of direct solar thermal absorption collectors using nanofluids. Several studies have attempted to mathematically model thermal conductivity enhancement, but few can fully explain the mechanisms behind this phenomenon and their subsequent contributions to the enhancement seen in nanofluids. The present work has also emphasized the relatively small number of articles discussing other nanofluid properties such as surface tension, photo-thermal response and viscosity. Furthermore, apart from thermal conductivity enhancement, very few modelling studies have been undertaken to predict nanofluid properties. Thus, the present review has not only highlighted the need to develop nanofluids, but also the imperative need to investigate their unique properties and evaluate their performance in direct solar thermal absorption collectors. Further research in this field is urgent, since the incorporation of nanofluids in direct solar thermal adsorption collectors can deliver an alternative, eco-friendly and viable renewable energy source. 

## Figures and Tables

**Table 1:** A selection of typical solar thermal collector configurations.

Motion		Collector Configuration			Concentration Ratio	Temperature Range (°C)	Reference
		Name/Absorber					
Stationary		Flat plate collector	FPC	Flat	C ≤ 1	30 ≤ T ≤ 80	[20]
		Evacuated tube collector	ETC		C ≤ 1	50 ≤ T ≤ 230	[21]
		Compound parabolic	CPC	Tubular	1 ≤ C ≤ 5	60 ≤ T ≤ 240	[22]
Sun Tracking	Single axis	Collector			5 ≤ C ≤ 15	60 ≤ T ≤ 290	
		Fresnel lens collector	FLC		10 ≤ C ≤ 40	60 ≤ T ≤ 270	[23]
		Parabolic trough collector	PTC		15 ≤ C ≤ 45	60 ≤ T ≤ 400	[24]
		Cylindrical trough collector	CTC		10 ≤ C ≤ 50	60 ≤ T ≤ 400	[25]
	Two axis	Spherical Bowl Reflector	SBR	Point	100 ≤ C ≤ 300	70 ≤ T ≤ 700	[26]
		Parabolic dish reflector	PDR		100 ≤ C ≤ 1000	100 ≤ T ≤ 900	[26]
		Heliostat field collector	HFC		100 ≤ C ≤ 1500	150 ≤ T ≤ 2000	[27]

**Table 2:** A selection of thermal conductivities of solid particles and working fluids at 25 °C.

Material	Thermal Conductivity (Wm^−1^K^−1^)	Reference
*Metals*		
Gold	315	[72]
Silver	424	[72]
Copper	398	[72]
Aluminum	273	[72]
Iron	80	[72]
Steel	46	[21]
Stainless Steel	16	[21]
*Metal Oxides*		
Alumina (Al_2_O_3_)	40	[73]
Cupric Oxide	77	[57]
Iron (II, III) Oxide	7	[74]
Titanium dioxide	8.37	[75]
Zinc Oxide	29	[76]
*Carbons*		
Amorphous Carbon	1.59	[77]
Diamond	900–2320	[77]
Carbon Nano-fibers	13	[78]
Carbon Nanotubes	2000	[79]
C_60_–C_70_ (Fullerenes)	0.4	[57]
Graphite	2000	[80]
*Working Fluids*		
Water	0.608	[81]
Ethylene Glycol	0.257	[72]

**Table 3:** A selection of thermal conductivity measurements from several nanofluid studies.

Nanoparticle	Particle Size (nm)	Working Fluid	Fraction	Thermal Enhancement (%)	Reference
*Metals*					
Ag	<100	Water	0.3–0.9 vol %	30 at 50 °C	[50]
Ag	100–500	Ethylene Glycol	0.1–1.0 vol %	18	[205]
Cu	50–100	Water	0.1 vol %	24	[206]
Cu	<10	Ethylene Glycol	0.01–0.05 vol %	41	[67]
Fe	10	Ethylene Glycol	0.1–0.55 vol %	18	[103]
*Metal Oxides*					
Al_2_O_3_	9	Water	2–10 vol %	29	[207]
Al_2_O_3_	28	Water/Ethylene Glycol	3–8 vol %	41	[100]
Al_2_O_3_	650–1000	Transformer oil	0.5–4 vol %	20	[208]
CuO	100	Water	7.5 vol %	52	[209]
TiO_2_	15	Water	0.5–5 vol %	30	[159]
SiO_2_	12	Ethylene Glycol	1–4 vol %	23	[210]
*Carbons*					
Carbon Black	190	Water	4.4–7.7 vol %	10 at 35 °C	[55]
carbon/graphene oxide	Not specified	Ethylene Glycol	0–0.06 wt %	6.47 at 40 °C	[211]
SWCNT	Dia. 10–50 Len. 0.3–10 µm	Diesel Oil	0.25–1 vol %	10–46	[212]
MWCNT	25 nm × 50 µm	Oil	1 vol %	150	[79]
MWCNT	Dia. 10 Len. 5–15 µm	Gum Arabic & Water	0.14-0.24 vol %	10	[213]

**Table 4:** A selection of nanofluid-based direct absorption solar collector performance studies.

Nanoparticle Size (nm)	Working Fluid	Fraction	Major Result of Study	Reference
Al_2_O_3_ Below 20	Pure water	1.0 vol %	Collector efficiency increases partially with increasing in particle size	[97]
Al_2_O_3_ 15	Distilled Water + Triton X-100	0.2 wt % 0.4 wt %	Collector efficiency at 0.2 wt % is 28.3% above working fluid of water. With surfactant efficiency was 15.63%	[241]
CuO 25 & 50	Deionized water (surfactant)	0.01, 0.02, 0.04, 0.1, 0.2 wt %	Good absorption while transmittance decreases with increasing nanoparticle size and mass fraction.	[242]
TiO_2_ ,10; Al_2_O_3_, 20 Ag, 50; Cu, 50, SiO_2_, 50	Texatherm Oil	0.1, 1.0, 2.0, 3.0 vol %	Collector outlet temperature and efficiency were improved using Ag, Cu, SiO_2_ and Al_2_O_3_, but not for TiO_2._	[45]
Graphite, 35	Texatherm Oil	0.05, 3.0, 5.0 vol %	5.0 vol % significantly improved thermal conductivity compare to Texatherm oil	[45]
CNT Dia. 10–20 nm Length 10–30 µm	Texatherm Oil	1.0 vol %	Minor improvement in thermal conductivity of base working fluid	[45]
CNT Dia. 10–20 nm Length 0.5–2 µm	Texatherm Oil	1.0 vol %	Minor improvement in thermal conductivity of base working fluid	[45]
Carbon nanomaterials	* Ionic Liquid (BMIM)BF_4_	0.005 wt % 0.01 wt %	Temperature range 20 to 145 °C Thermal conductivity performance	[243]
Graphite 30	(BMIM)BF_4_	0.005 wt % 0.01 wt %	1.0 to 3.4% 9.4 to 10.7%	[243]
SWCNT, Dia. 2 nm Length 5 to 30 µm	(BMIM)BF_4_	0.005 wt % 0.01 wt %	6.2 to 5.8% 14.7 to 14.7%	[243]
Graphene 0.8 nm single layer Range: 0.5 to 2 µm	(BMIM)BF_4_	0.005 wt % 0.01 wt %	13.9 to 14.5% 16.3 to 15.4% Carbons also displayed significant temperature enhancement compared to base working fluid	[243]
Functionalized CNT	Deionized water	150 ppm	Thermal conductivity enhancement of 32.2% Also displayed good optical properties	[188]

* Ionic Liquid: 1-Butyl-3-methylimidazolium tetra fluoroborate.
